# Attitudes of Mental Health Professionals towards Telepsychology during the Pandemic: A Pilot Study

**DOI:** 10.3390/healthcare11111542

**Published:** 2023-05-25

**Authors:** Marco Cavallo, Elisa Pedroli, Manuela Cantoia, Breeda McGrath, Sonja Cecchetti

**Affiliations:** 1 Faculty of Psychology, eCampus University, 22060 Novedrate, CO, Italy; elisa.pedroli@uniecampus.it (E.P.); manuela.cantoia@uniecampus.it (M.C.); cecchettisonja@gmail.com (S.C.); 2 Clinical Psychology Service, Saint George Foundation, 12030 Cavallermaggiore, CN, Italy; 3 Applied Technology for Neuropsychology Lab, IRCCS Istituto Auxologico Italiano, 20135 Milan, MI, Italy; 4 The Chicago School of Professional Psychology, Chicago, IL 60601, USA; bmcgrath@thechicagoschool.edu

**Keywords:** attitudes, internet-based intervention, remote consultation, survey, telehealth, telepsychology

## Abstract

Objective: This pilot study investigated mental health professionals’ attitudes towards remote psychological consultations and internet-based interventions. Methods: An online survey in Italian and English was administered to a sample of 191 psychologists and psychotherapists to collect detailed information about their professional experience providing online psychological interventions a year and a half after the beginning of the SARS-CoV-2 pandemic. Results: The results did not reveal a statistically significant association between the participants’ theoretical approaches and the number of patients treated via the online modality. Overall, most of the participants found advantages to the online setting but also noted critical issues regarding privacy and the ease of integrating new technology into their clinical practice. Conclusions: According to the participants, despite the challenges that must be addressed, telehealth is a viable psychological therapeutic option that is destined to grow in importance in the near future.

## 1. Introduction

The global pandemic of Severe Acute Respiratory Syndrome Coronavirus-2 (SARS-CoV-2) [1] caused a state of emergency that exacerbated existing difficulties in accessing basic services, such as treatment for mental disorders [2,3,4]. Although in-person psychological interventions were still available during the pandemic [5], the World Health Organization drew up a set of guidelines to contain the spread of SARS-CoV-2 [6], and the United Nations recommended transferring in-person activities such as mental health services to online modalities where possible [7].

The American Psychological Association (APA) [8] defines telepsychology as the provision of psychological services using telecommunication technologies.

Although research interest in telepsychology was already evident before the pandemic [9,10], it was not commonly used in standard clinical practice before this universal crisis [11]. As with all mental health treatments, the provision of online psychological interventions through digital technology must comply with ethical and safety requirements. However, telepsychology includes idiosyncrasies that are dependent on the computer literacy skills of the mental health professional, which can promote trust in or inhibit the use of such treatment [12,13].

A number of studies surveyed mental health professionals between the end of 2019 and the end of 2020, with the aim of investigating the transition from face-to-face psychological interventions to telepsychology [5,14]. The survey data provided some promising results, and it should be noted that telepsychology interventions have sometimes led to creative adaptations in the ways in which therapies are delivered and supervised [15,16]. For example, Downing et al. [17] surveyed Australian psychologists’ use of telepsychology during the COVID-19 crisis, gathering evidence to highlight potential shortcomings, such as the limited viewing area of a screen that reduces therapists’ capacity to read and respond to their patient’s body language, and the ‘distortion’ of the therapeutic atmosphere typically created in face-to-face therapy. Other studies, on the other hand, aimed to investigate whether specific therapeutic techniques, such as the chair technique, tele-chairwork [18], remote mindfulness [19,20], and internet-mediated eye movement desensitisation and reprocessing (EMDR) [21], could be applied fruitfully in the remote context. In addition, Fogler et al. [22] adapted and administered a remote version of a brief parent training group targeting caregivers of school-aged children with ADHD. Both clinicians and participants rated the methodological appropriateness and clinical effectiveness of the intervention positively, showing that telepsychology in this field can be used with equal levels of process fidelity and satisfaction in face-to-face interventions.

Moreover, McKee et al. [23] investigated psychologists’ telepsychology use during the pandemic in a large sample of US-licensed psychologists. Their results showed that psychologists’ attitudes concerning telepsychology are associated with intentions to use telepsychology, which in turn has an impact on the amount of clinical work they actually perform remotely. In addition, the perception of the usefulness of the online setting, combined with the ease of use of technology tools, is associated with participants’ attitudes towards telepsychology.

Similar to the theory that claims that exposure to certain objects leads to familiarity [24], it could be hypothesised that repeated and intense exposure to new technologies may lead to familiarity, which allows greater comfort during use.

According to Perle and others, it is of paramount importance to invest in telehealth training, e.g., [25]. Otherwise, as technological progress continues, the use of the remote mode could worsen the workload, fatigue, and burnout of mental health professionals [26].

As can be seen from a number of systematic reviews, most of the scientific literature focuses on cognitive–behavioural orientation [27,28], and some authors have indicated the scarcity of research on different theoretical approaches for telepsychological interventions [29].

Despite the interesting data available, studies published to date do not address whether telepsychology interventions are effective irrespective of the practitioners’ therapeutic orientation. The first aim of the present pilot study was therefore to clarify whether there is an association between health professionals’ therapeutic orientation and their satisfaction with remote clinical psychology practice. The additional aims were also to investigate the methodological and applied practice advantages and disadvantages of telepsychology during the pandemic.

## 2. Materials and Methods

### 2.1. Study Design

This study adopted a mixed-methods approach aimed at collecting in-depth information from practitioners about their perspectives on online psychological interventions experienced during the SARS-CoV-2 pandemic. This study examined quantitative and qualitative data (QUAN + QUAL) in parallel, so that the data performed complementary functions [30]. Data were collected through an online survey [31], which enabled ad hoc and remote data collection. This study design presented several advantages, such as speed of execution, a low cost for implementation, and immediacy of results [32].

### 2.2. Participants

A total of 204 participants signed the informed consent form to begin the survey, declaring that they were qualified psychologists and/or psychotherapists. The exclusion criterion was a lack of experience with remote psychological assessment and treatment. Therefore, those who answered negatively to this item were not allowed to continue with the questionnaire. A total of 12 participants were excluded from the research, and 191 were included, which was a sufficiently large number on the basis of a priori statistical power calculations to suggest that the results can be generalised to a larger body of professionals.

### 2.3. Procedure

Data collection was conducted online during the period from 5 September 2021 to 28 January 2022. The research team recruited mental health professionals who identified as psychologists or psychotherapists, who had experience providing online interventions to their patients. To identify potential participants in the survey, snowball sampling and purposive sampling methods were used across social media platforms such as LinkedIn, Facebook, and Instagram, as main sources. These social media platforms contain the professional profiles of many self-defined psychologists or psychotherapists. Several recent studies have used social media similarly to identify and recruit samples for telepsychotherapy research, e.g., [33]. The authors also used their own personal networks to identify potential survey participants and sent email invitations to researchers, relevant stakeholders, rehabilitation centres, research centres, and organisations operating digital platforms for psychologists (please see the Supplementary Material in Table S1 for more information about the participants).

Participation in the survey was voluntary, and no incentives were provided to participants. All participants signed informed consent forms before starting the survey. The data were collected and processed in an anonymous and aggregated form in accordance with the current EU GDPR (2016, pd. 196/03).

### 2.4. Measures

The research was conducted through a web survey on the Google Forms platform and was made accessible through a live Internet link. The survey was designed according to best practices in the recent literature [33,34,35]. The online questionnaire was available in both Italian and English and consisted of 62 items in total. The questionnaire included both closed- and open-ended items aimed at investigating the following specific thematic areas: “Online experience”, “Professional profile”, “Personal data”, “Clinical work”, “Online setting”, “Online assessment and testing”, “New technologies”, “Performance compensation”, and “Perspectives”. The closed-ended items included a Likert scale and dichotomous and multiple-choice responses. Numerous free-text comments of the survey participants provided confirmation that the selection criteria were solidly met (please see Supplementary Material file S1 for the English version of the survey questionnaire).

### 2.5. Data Analyses

All data analyses were performed using the statistical software R (v. 4.0.3) [36]. After assessing the demographic characteristics of the sample, data analyses of the closed-ended items were conducted to formulate frequency tables of each variable, construct contingency tables between two variables, and investigate possible significant associations.

In order to fulfil the objectives of the research study, we examined whether a relationship exists between the variables of item no. 4 (mental health professional’s therapeutic approach) and item no. 15 (number of patients/clients seen online weekly). A Pearson’s chi-squared test for the independence of categorical variables was performed, and a possible association with Cramér’s V [37] between nominal variables was quantified.

Regarding non-parametric ordinal variables, we checked Kendall’s tau-b correlation coefficient to explore a possible association between the variables in items 13 and 15, and we also investigated a relationship between the variables in items 52 and 27. This non-parametric test was chosen because it does not require distributional normality of the variables, nor that the relationship be linear, and it is also more robust to outliers. The association estimate calculated with this index is more accurate even if the sample size is small. For graphic visualisation of the data, most of the figures were created with the installation of the library ‘tydeverse’ and the function ggplot2.

Text-mining analyses were conducted only on the open-ended to analyse the advantages and critical features of the online setting, and two different word clouds were created using “tm”, “SnowballC”, “wordcloud”, and “RcolourBrewer” software to highlight the frequency of the words reported by the respondents using colours and sizes.

## 3. Results

The results presented below reflect the responses of the 191 participants and are expressed as the number (n) and the percentage (%) of respondents. A description of the sample and comparisons between participants are reported in Table 1.

### 3.1. Clinical Activity

Examining the respondents’ motivations for engaging in online psychological treatment, the data revealed that distance was mentioned in 73% of cases, and the prevention of SARS-CoV-2 infection was mentioned in 50%.

The majority of psychotherapists/psychologists agreed that having previous in-person clinical experience can be very helpful for online clinical practice. On the Likert scale of 1 to 5, where 1 = not at all and 5 = very much, 6% answered 2, 16% answered 3, 31% answered 4, and 47% answered 5 (very much), (please see Supplementary Material Figures S1 and S2 for more information about their clinical experience).

In order to better prepare themselves for online clinical practice, the majority of professionals (73%) read guidelines; scientific articles (44%); asked colleagues for advice (46%); and attended webinars (45%). Of all respondents, 60% felt that their academic training was not adequate for online therapy, whereas the majority (61%) claimed that refresher courses were adequate.

The majority (72%) felt that the adherence of clients to online therapy was equivalent to that of face-to-face therapy. Of the respondents, 45% felt that the online modality does not lead to a greater rate of drop-out, whereas 19.4% believed that it does.

**Figure 1:**
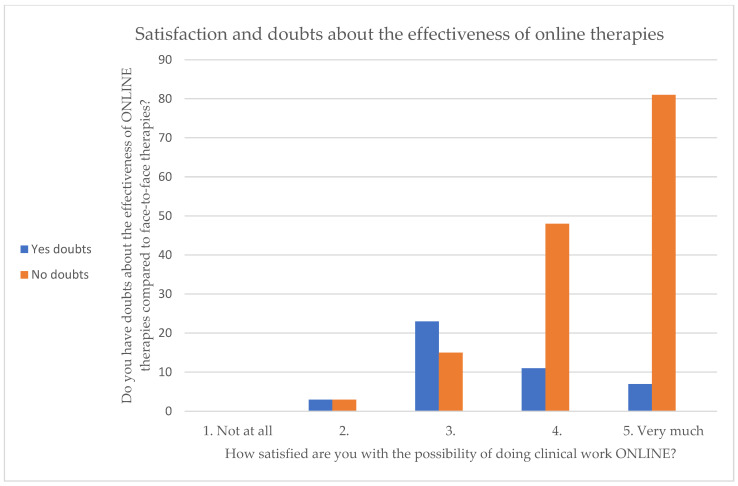
Satisfaction with online therapies (on a Likert scale ranging from 1—“Not at all” to 5—“Very much”).

On the Likert scale of 1 to 5 regarding satisfaction with practising their profession online, no respondents answered 1 (not at all), whereas 46% indicated a 5 (very much). The majority of the respondents stated that they have confidence in the effectiveness of online therapy. Other interesting results emerge when observing the joint distribution of the degree of satisfaction in being able to conduct therapy online (2–5) and of having doubts as to whether this modality is effective (dichotomous variable). Those who gave the highest scores in satisfaction showed the fewest doubts about effectiveness (Figure 1).

### 3.2. Associations between Variables

The first research objective was to investigate the presence of a relationship between therapeutic orientation and the promotion of online psychological treatment, expressed by the number of online clients per week. The chi-squared test (X-squared = 22.406, Df = 24, *p*-value > 0.05) showed that there is no association between the two variables. This indicates that no discrimination was detected between treatment orientations towards the practice of online therapies.

In addition, we were also interested in investigating the presence of a possible association between the number of years of online clinical practice (item 13) and the number of online patients (item 15). As the two variables are ordinal, they were analysed using Kendall’s tau-b correlation coefficient (Z = 4.0794, tau = 0.2629779, *p*-value < 0.05), leading us to reject the null hypothesis of no correlation. The tau of 0.26 indicated a slight positive concordance between the two variables. This positive concordance suggests that, as the years of online experience increase, the number of patients using this modality also tends to increase.

The third possible association between the degree of satisfaction in carrying out online therapy (item 52) and perceived work fatigue (item 27) was also explored. As the two variables are ordinal, they were also studied with Kendall’s tau-b (Z = −3.1983, tau = −0.2077453, *p*-value < 0.05). Here, too, the null hypothesis (no association) was rejected, and with a tau of −0.21, a slight negative concordance was revealed. The presence of a negative concordance indicates an inverse association; as perceived online fatigue becomes higher, the level of satisfaction becomes lower. Please see the Supplementary Materials for additional correlations between demographic and clinical variables.

### 3.3. Telepsychological Interventions

A description of the telepsychological interventions and the comparisons between the participants are illustrated in Table 2.

More than half of the participants (58.7%) indicated that most of their online patients were seen online first; 22.8% indicated that they were followed in a mixed modality (both in-person and online); and 18.5% indicated that most of their patients who were already in treatment switched from in-person to online.

Among the categories of patients treated online, children appeared to be the least frequently seen, and those treated online least often were groups and families. People with anxiety disorders were treated with online clinical practice by almost all the participants interviewed, whereas people with cognitive disorders were the least often served online.

### 3.4. Online Assessment and Testing

In most cases, the participants administered tests remotely using a video call, a platform for self-administration, or screen sharing, whereas some did not use any. Only 3% of the participants used online neuropsychological assessment.

### 3.5. New Technologies

Despite the range of choices in the item of preferred devices for conducting telepsychological interventions, 70% of participants chose a PC. The most frequently used mode of interaction with patients in telepsychology was videoconferencing (93%). In addition, the majority of respondents stated that they use Skype (65%), WhatsApp (42%), and Zoom (41%), despite the option to select additional answers. Regarding the reasons for preferring one software over others, the majority of participants (87%) indicated ease of use. The most commonly used connection was Broadband, chosen by 47% of the sample.

Most respondents had a personal website (64%), but only a minority had a corporate website (35%). The social networks most used to create a professional profile accessible to attract new patients were LinkedIn (70%), Facebook (72%), and Instagram (73%). The most frequently used booking methods for telepsychological interventions were telephone calls and e-mails (please see Supplementary Material Table S2 for more information about the new technologies used).

### 3.6. Fees for Services

**Figure 2:**
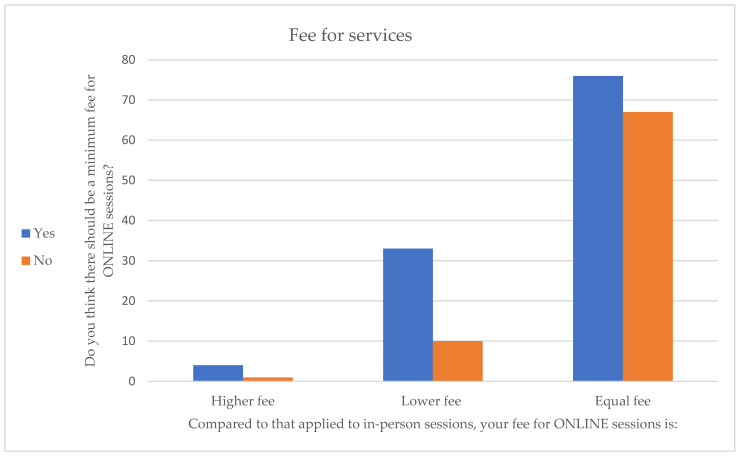
Fees for services.

Regarding the fee schedule, most of the psychotherapists and psychologists who participated in the research stated that their fee was the same as that for the face-to-face modality, whereas 23% charged a lower rate. The majority of participants (59%) claimed that there is a need for a minimum fee for online sessions. Those who maintained the same fee schedule for the two modalities were divided between whether or not there is a need for a minimum online fee schedule, whereas those who charged a lower price in the online modality were more likely to affirm the need for a minimum fee schedule (Figure 2). Of the participants, 46.6% stated that they received the session fee afterwards, whereas 18% stated that they received it in advance. The majority of respondents (65%) did not feel that there is a difference between the risk of default in payment between online and face-to-face sessions.

### 3.7. Advantages and Criticalities

Although 66% of the participants found advantages in the online setting, 54% also found critical or negative aspects compared to the traditional face-to-face setting.

**Figure 3:**
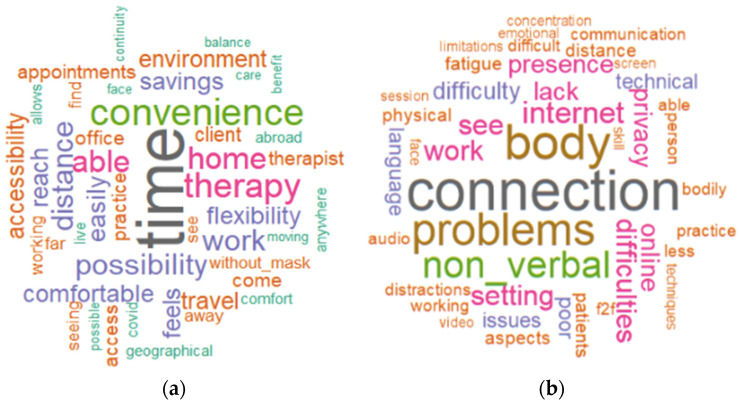
Advantages (**a**) and criticisms (**b**) of the online setting.

In this last part of the analysis, we focused on two open-ended questions that were posed to the participants. We created a word cloud for each of the two items to highlight the most frequently used terms (Figure 3). The word cloud depicts in the largest font the words most frequently used by the participants. The criteria set for the analysis of answers about the benefits of online therapy had a minimum frequency of 3 and a maximum of 100 words. The words that occurred most frequently were as follows: time (32 responses), convenience (17 responses), flexibility (9 responses), comfort (8 responses), and accessibility (8 responses). This suggests that the perceived advantages are those that make it possible to work in a more comfortable and optimised manner.

We then analysed all the answers concerning the most frequent criticisms of the online setting. A minimum frequency of 3 and a maximum number of 100 words were required for this analysis. The critical words that occurred most frequently were as follows: connection (30 responses), body (22 responses), non-verbal (15 responses), difficulties (10 responses), privacy (8 responses), and distractions (6 responses). Overall, the problems encountered in conducting online therapy are more attributable to technical problems than to the practitioner’s ability to carry out therapeutic work at a distance, although difficulties related to the lack of bodily presence and the reduced scope of non-verbal language also emerged.

## 4. Discussion

Although telepsychology represents a challenge to the therapeutic setting and relationship [39,40,41,42], it has been possible to accelerate the process of digitising the psychology profession that had already begun in some countries where communities have significant problems with access to care [43,44].

The first aim of the present pilot study was to clarify whether there is an association between health professionals’ therapeutic orientation and their satisfaction with remote clinical psychology practice. The additional aims were also to investigate the methodological and applicative advantages and disadvantages of remote psychology practice during the pandemic, as perceived by mental health professionals.

No significant associations emerged in the present study between the therapeutic approach of the mental health professionals and the promotion of online psychological treatment, as expressed by the number of online patients seen per week. Although the belief that carrying out clinical activity online is easier than or of equal difficulty to in-person practice, as detected among younger professionals, some authors warn against this assumption, calling for it to be replaced with a cautious and prudent attitude [45]. In this regard, the present research found that 47% of respondents agreed that having previous clinical experience in-person can be very useful in online clinical practice as well. Keeping with this, our study found a positive association between the number of years of online clinical practice and the number of online patients. This positive association indicates that, as the number of years of online experience increased, the number of patients attending sessions remotely also tended to increase. Thus, within our sample, online psychological interventions were an important working tool even before the pandemic for a significant proportion of participants. The present study also investigated the respondents’ perceived degree of satisfaction with telepsychology and found that more than half (56%) of participants answered 5 (very much) on a Likert scale of 1 to 5. Those who gave the highest scores in satisfaction showed fewer doubts as to its effectiveness. In this regard, the present research showed a slightly negative correlation between the degree of satisfaction with online therapies and perceived work fatigue.

A recent APA [46] COVID-19 Telehealth practitioner survey revealed that the prolonged pandemic continued to have an impact on mental health treatment and demand. The majority of respondents to the survey reported an increase over the previous year in online adult (46%) and adolescent (43%) patients, compared to lower proportions of children (24%) and older adults (14%). Consistent with these results, the participants in the present study stated that they mainly treated adult patients online (97%), with fewer but still an appreciable frequency of adolescents (42%) and, to a much lesser extent, children (10%) and older adults (28%). Although the scientific literature indicates that online psychological treatments are also suitable for children, they may have a greater need for the actual physical presence of a therapist while participating in therapy, in part due to greater difficulty paying attention for long periods of time, especially if in the presence of distracting stimuli. Regarding their clinical work, the recruited mental health professionals stated that they treated mainly people with anxiety (94%), depressive disorders (84%), and PTSD (47.1%) less frequently. The results of our study are consistent with the results reported in the APA survey [46,47]. Although a significant proportion of the participants stated that they started to use remote psychological treatments due to the pandemic, a large number were already familiar with online therapeutic treatment options.

Regarding the types of therapies delivered online, all participants in this sample stated that they deliver individual psychological treatments (100%), whereas markedly fewer deliver couple (26%) and family (16%) treatments as well. These low reported frequencies of online couple and family therapies are consistent with previously reported rates [48,49]. The online modality with multiple patients may be more challenging due to the factors involved, such as the complex dynamics of the group as a whole. Prior training of therapists and patients would likely be essential in a move from face-to-face to online groups [34,40].

Regarding the psychological assessment, several authors concur and support the robust evidence that clinical interviews conducted through online assessment procedures are equivalent to traditional in-person procedures [50,51,52,53,54]. However, although several studies have suggested that the quality of data collected through online assessments is comparable to that of traditional in-person procedures, especially for structured clinical interviews [51,55,56,57] and forensic evaluations [58,59], 27% of the participants indicated that they did not administer tests remotely. Although the scientific literature also highlights the efficacy of online neuropsychological assessment based on the assessment of older adults with and without cognitive impairment [60,61,62,63,64,65,66,67], only a minority of respondents in this study indicated that they conduct online neuropsychological assessments. In the same vein, the present research suggests that online assessments may represent a weakness that has yet to be addressed adequately.

Regarding the important topic of service fees, the results showed that the majority of participants kept their treatment fees unchanged from that of the face-to-face setting, whereas 23% charged a lower rate. In addition, most of the participants (59%) claimed that there is a need for a standard minimum fee for online sessions. A minority of participants stated that they charge less for online compared to face-to-face sessions, and only a very limited proportion support the need of higher fees for remote consultations.

The telepsychology modality used by most of the participants (93%) in this study was videoconferencing (93%). This finding is consistent with results reported by the APA [46]. The majority of the mental health professionals who participated in this study stated that they prefer Skype (65%) as the software for videoconferencing, similar to the findings reported by Humer and colleagues [14].

The results of the present study show that clinicians critique the online setting or modality due to weaknesses in connection and privacy protection. This is consistent with recent findings in an APA survey [46], in which connectivity problems were cited by 55% of respondents, and 50% indicated privacy problems.

Regarding future perspectives, 92.1% of respondents in this study claimed they will continue to use online consultations in the future. This finding is concordant with other studies indicating that many therapists expect online clinical work to become a substantial part of their practice in the future [33,68]. The results also show that the majority of the participants (60%) felt that their academic training is not adequate for the demands of online therapy. Similarly, other authors believe that telepsychology should soon become a central topic in psychotherapy training [33], and it will be crucial to continue investing in research in this field [25,69].

This study has some limitations. A first limitation is the fact that the survey was conducted online. Although a web-based survey is an efficient, flexible, and inexpensive method that allows for the recruitment of a relatively large sample [40,70], it may have led to some biases, such as a higher percentage of participants with a predilection for new technologies [27]. In addition, a significant proportion of participants were less than 40 years of age, which is not fully representative of the entire population of psychologists and psychotherapists. This narrow age sample and potential bias may have contributed to the unexpectedly large percentage who reported a positive evaluation of online psychological treatment [71]. Conducting the survey online may have also caused a selection bias towards those who are already familiar with and who invested in the use of technology, resulting in a smaller number of older participants [72]. Lastly, as the survey was translated in Italian and English only, our pilot study was not available to mental health professionals who were not familiar with these two languages.

Regarding the strengths of this study, the sample is appropriate in size (as reflected in the a priori statistical power analysis calculations), thus indicating that the participants in this study were likely to be representative of a wider population of mental health practitioners. In addition, we were able to reach a large number of psychologists and psychotherapists of different therapeutic approaches, allowing us to include a plurality of clinical points of view.

## 5. Conclusions

In conclusion, this study provides important insights into mental health professionals’ experiences, evaluations, and expectations about telepsychological interventions and their integration into therapeutic treatment. In the future, we may expect a mix of both face-to-face and remote services. However, as participants indicated, the current models of academic and practical training for psychologists and psychotherapists in online therapy are not adequate. To better prepare themselves for online clinical practice, the majority of the professionals recruited relied on practice guidelines, scientific articles, collegial advice, and online lectures or webinars. Although individual professional development and continuing education is commendable, a common and shared perspective on formal and substantial training in this area of practice is still essential. Thus, in the future, it will be imperative for academic programs to include specific training on how to provide and evaluate online psychological interventions. Mental health professionals should acquire additional skills to leverage new technological opportunities to increase access to services, while at the same time maintaining an appropriately cautious attitude to manage potential risks. The long-term effects of telepsychological interventions—particularly related to perceived satisfaction and burn-out—should be investigated further. In addition to mental health professionals’ views, patients’ perspectives should also be evaluated and included in training considerations and planning. The ultimate goal for mental health professionals is to provide competent, caring treatment for their patients, finding the best way forward for each individual, couple, family, or group.

## Figures and Tables

**Table 1:** Descriptive statistics of the 191 surveyed participants.

Variables	n	%
Gender		
Female	104	54.5
Male	86	45.0
Non-binary	1	0.5
Age (years)		
<30	36	18.8
30–39	83	43.5
40–49	34	17.8
50–59	22	11.5
60–69	12	6.3
>69	4	2.1
Professional qualification		
Psychotherapist	115	60.0
Psychologist	76	40.0
Private	167	87.2
Public	16	8.5
Affiliates	8	4.3
Years of online clinical experience		
<1	39	20.4
1–3	111	58.1
4–6	23	12.0
7–9	8	4.2
>9	10	5.2
Number of online patients in a week		
1–5	114	59.7
6–10	36	18.8
11–20	21	11.0
21–30	13	6.8
>30	7	3.7
Therapeutic approach *		
Psychoanalysis and psychodynamic therapies	37	19.4
Behavioural therapy	50	26.2
Cognitive therapy	25	13.1
Humanistic therapy	21	11.0
Integrative and holistic therapy	41	21.5
Systemic therapy	15	7.9
None	2	1.0

* Data are aggregated according to the therapeutic approaches recognised by the APA Encyclopedia of Psychology [38] with the addition of systemic therapy.

**Table 2:** Descriptive statistics about telepsychological interventions.

Variables	n	%
Among the patients you receive online are *:		
Children	19	9.9
Adolescents	80	41.9
Adults	186	97.4
Elderly	53	27.7
What types of telepsychological interventions do you carry out online *:		
Individual	191	100
Couple	53	27.7
Family	30	15.7
Group	27	14.1
Your online psychology treatments concern *:		
Anxiety disorders	180	94.2
Depressive disorders	161	84.3
PTSD	90	47.1
Cognitive disorders	55	28.8

Note. * The asterisk indicates that multiple answers were possible per respondent.

## Data Availability

Raw data supporting the conclusions of this article will be made available by the authors upon request.

## Supplementary Materials

The following supporting information can be downloaded at: https://www.mdpi.com/article/10.3390/healthcare11111542/s1, Table S1: Geographical distribution of mental health professionals included in this study; Table S2: Descriptive statistics about the new technologies used; Table S3: Association between the professionals’ degree of satisfaction and their therapeutic approach; Figure S1: Clinical experience and online clinical experience of the sample of mental health professionals; Figure S2: Distributions of patients received face-to-face per week and patients received online per week by the sample of mental health professionals; Supplementary material file S1: The full survey (in English) used in the present study.

## References

[1] Gorbalenya A.E., Baker S.C., Baric R.S., de Groot R.J., Drosten C., Gulyaeva A.A., Haagmans B.L., Lauber C., Leontovich A.M., Neuman B.W. (2020). The species Severe acute respiratory syndrome-related coronavirus: Classifying 2019-nCoV and naming it SARS-CoV-2. Nat. Microbiol..

[2] Brooks S.K., Webster R.K., Smith L.E., Woodland L., Wessely S., Greenberg N., Rubin G.J. (2020). The psychological impact of quarantine and how to reduce it: Rapid review of the evidence. Lancet.

[3] Rajkumar R.P. (2020). COVID-19 and mental health: A review of the existing literature. Asian J. Psychiatr..

[4] Wang C., Pan R., Wan X., Tan Y., Xu L., Ho C.S., Ho R.C. (2020). Immediate Psychological Responses and Associated Factors during the Initial Stage of the 2019 Coronavirus Disease (COVID-19) Epidemic among the General Population in China. Int. J. Environ. Res. Public. Health.

[5] Boldrini T., Schiano Lomoriello A., Del Corno F., Lingiardi V., Salcuni S. (2020). Psychotherapy During COVID-19: How the Clinical Practice of Italian Psychotherapists Changed During the Pandemic. Front. Psychol..

[6] World Health Organization Coronavirus Disease (COVID-19) Technical Guidance: Infection Prevention and Control/WASH. https://www.who.int/emergencies/diseases/novel-coronavirus-2019/technical-guidance/infection-prevention-and-control.

[7] United Nations United Nations Policy Brief: COVID-19 and the Need for Action on Mental Health. https://www.un.org/sites/un2.un.org/files/un_policy_brief-covid_and_mental_health_final.pdf.

[8] American Psychological Association Guidelines for the Practice of Telepsychology. https://www.apa.org/practice/guidelines/telepsychology.

[9] Mitchell E. (2020). “Much more than second best”: Therapists’ experiences of videoconferencing psychotherapy. Eur. J. Qual. Res. Psychother..

[10] Vis C., Kleiboer A., Prior R., Bønes E., Cavallo M., Clark S.A., Dozeman E., Ebert D., Etzelmueller A., Favaretto G. (2015). Implementing and up-scaling evidence-based eMental health in Europe: The study protocol for the MasterMind project. Internet Interv..

[11] Probst T., Haid B., Schimböck W., Reisinger A., Gasser M., Eichberger-Heckmann H., Stippl P., Jesser A., Humer E., Korecka N. (2021). Therapeutic interventions in in-person and remote psychotherapy: Survey with psychotherapists and patients experiencing in-person and remote psychotherapy during COVID-19. Clin. Psychol. Psychother..

[12] Ahlström K., von Below C., Forsström D., Werbart A. (2022). Therapeutic encounters at the onset of the COVID-19 pandemic: Psychodynamic therapists’ experiences of transition to remote psychotherapy. Psychoanal. Psychother..

[13] Mendes-Santos C., Weiderpass E., Santana R., Andersson G. (2020). Portuguese Psychologists' Attitudes Toward Internet Interventions: Exploratory Cross-Sectional Study. JMIR Ment. Health.

[14] Humer E., Stippl P., Pieh C., Schimböck W., Probst T. (2020). Psychotherapy via the Internet: What Programs Do Psychotherapists Use, How Well-Informed Do They Feel, and What Are Their Wishes for Continuous Education?. Int. J. Environ. Res. Public Health.

[15] Tarlow K.R., McCord C.E., Nelon J.L., Bernhard P.A. (2020). Comparing in-person supervision and telesupervision: A multiple baseline single-case study. J. Psychother. Integr..

[16] Waller G., Pugh M., Mulkens S., Moore E., Mountford V.A., Carter J., Wicksteed A., Maharaj A., Wade T.D., Wisniewski L. (2020). Cognitive-behavioral therapy in the time of coronavirus: Clinician tips for working with eating disorders via telehealth when face-to-face meetings are not possible. Int. J. Eat. Disord..

[17] Downing L., Marriott H., Lupton D. (2021). “‘Ninja’ levels of focus”: Therapeutic holding environments and the affective atmospheres of telepsychology during the COVID-19 pandemic. Emot. Space Soc..

[18] Pugh M., Bell T., Dixon A. (2021). Delivering tele-chairwork: A qualitative survey of expert therapists. Psychother. Res..

[19] Duran É.P., Hemanny C., Vieira R., Nascimento O., Machado L., de Oliveira I.R., Demarzo M. (2022). A Randomized Clinical Trial to Assess the Efficacy of Online-Treatment with Trial-Based Cognitive Therapy, Mindfulness-Based Health Promotion and Positive Psychotherapy for Post-Traumatic Stress Disorder during the COVID-19 Pandemic: A Study Protocol. Int. J. Environ. Res. Public Health.

[20] Hutchison M., Russell B.S., Gans K.M., Starkweather A.R. (2022). Online administration of a pilot mindfulness-based intervention for adolescents: Feasibility, treatment perception and satisfaction. Curr. Psychol..

[21] Bursnall M., Thomas B.D., Berntsson H., Strong E., Brayne M., Hind D. (2022). Clinician and Patient Experience of Internet-Mediated Eye Movement Desensitisation and Reprocessing Therapy. J. Psychosoc. Rehabil. Ment. Health.

[22] Fogler J.M., Normand S., O’Dea N., Mautone J.A., Featherston M., Power T.J., Nissley-Tsiopinis J. (2020). Implementing Group Parent Training in Telepsychology: Lessons Learned During the COVID-19 Pandemic. J. Pediatr. Psychol..

[23] McKee G.B., Pierce B.S., Donovan E.K., Perrin P.B. (2021). Examining models of psychologists' telepsychology use during the COVID-19 pandemic: A national cross-sectional study. J. Clin. Psychol..

[24] Marks L.J., Olson J.C. (1981). Toward a cognitive structure conceptualization of product familiarity. ACR North Am. Adv..

[25] Perle J.G., Perle A.R., Scarisbrick D.M., Mahoney J.J. (2022). Educating for the Future: A Preliminary Investigation of Doctoral-Level Clinical Psychology Training Program's Implementation of Telehealth Education. J. Technol. Behav. Sci..

[26] Hilty D.M., Armstrong C.M., Smout S.A., Crawford A., Maheu M.M., Drude K.P., Chan S., Yellowlees P.M., Krupinski E.A. (2022). Findings and Guidelines on Provider Technology, Fatigue, and Well-being: Scoping Review. J. Med. Internet Res..

[27] Markowitz J.C., Milrod B., Heckman T.G., Bergman M., Amsalem D., Zalman H., Ballas T., Neria Y. (2021). Psychotherapy at a Distance. Am. J. Psychiatry.

[28] Poletti B., Tagini S., Brugnera A., Parolin L., Pievani L., Ferrucci R., Compare A., Silani V. (2021). Telepsychotherapy: A leaflet for psychotherapists in the age of COVID-19. A review of the evidence. Couns. Psychol. Q..

[29] Moshe I., Terhorst Y., Philippi P., Domhardt M., Cuijpers P., Cristea I., Pulkki-Råback L., Baumeister H., Sander L.B. (2021). Digital interventions for the treatment of depression: A meta-analytic review. Psychol. Bull..

[30] Tong A., Sainsbury P., Craig J. (2007). Consolidated criteria for reporting qualitative research (COREQ): A 32-item checklist for interviews and focus groups. Int. J. Qual. Health Care.

[31] Couper M. (2008). Designing Effective Web Surveys.

[32] Grimes D.A., Schulz K.F. (2002). An overview of clinical research: The lay of the land. Lancet.

[33] McBeath A.G., du Plock S., Bager-Charleson S. (2020). The challenges and experiences of psychotherapists working remotely during the coronavirus pandemic. Couns. Psychother. Res..

[34] Cipolletta S., Mocellin D. (2018). Online counseling: An exploratory survey of Italian psychologists’ attitudes towards new ways of interaction. Psychother. Res..

[35] Aafjes-van Doorn K., Békés V., Prout T.A., Hoffman L. (2020). Psychotherapists' vicarious traumatization during the COVID-19 pandemic. Psychol. Trauma.

[36] R Core Team R: A Language and Environment for Statistical Computing (Version 4.0. 3) [Computer Software]. R Foundation for Statistical Computing. https://www.r-project.org.

[37] Cramér H. (1946). Mathematical Methods of Statistics.

[38] American Psychological Association Different Approaches to Psychotherapy. https://www.apa.org/topics/psychotherapy/approaches.

[39] Aafjes-van Doorn K., Békés V., Prout T.A. (2021). Grappling with our therapeutic relationship and professional self-doubt during COVID-19: Will we use video therapy again?. Couns. Psychol. Q..

[40] Békés V., Aafjes-Van Doorn K. (2020). Psychotherapists’ attitudes toward online therapy during the COVID-19 pandemic. J. Psychother. Integr..

[41] Crowe M., Inder M., Farmar R., Carlyle D. (2021). Delivering psychotherapy by video conference in the time of COVID-19: Some considerations. J. Psychiatr. Ment. Health Nurs..

[42] Messina I., Loffler-Stastka H. (2021). Psychotherapists' perception of their clinical skills and in-session feelings in live therapy versus online therapy during the COVID-19 pandemic: A pilot study. Res. Psychother..

[43] McGinty K.L., Saeed S.A., Simmons S.C., Yildirim Y. (2006). Telepsychiatry and e-Mental Health Services: Potential for Improving Access to Mental Health Care. Psychiatr. Q..

[44] Schopp L.H., Demiris G., Glueckauf R.L. (2006). Rural backwaters or front-runners? Rural telehealth in the vanguard of psychology practice. Prof. Psychol. Res. Pract..

[45] Algeri D., Gabri S., Mazzucchelli L. (2018). Consulenza Psicologica Online: Esperienze Pratiche, Linee Guida e Ambiti di Intervento.

[46] American Psychological Association Worsening Mental Health Crisis Pressures Psychologist Workforce. 2021 COVID-19 Telehealth Practitioner Survey..

[47] American Psychological Association Psychologists Embrace Telehealth to Prevent the Spread of COVID-19. A Survey of APA Members Reveals How the Pandemic Has Impacted Clinicians and Their Practices. https://www.apaservices.org/practice/legal/technology/psychologists-embrace-telehealth?_ga=2.31554750.1059429488.1663668573-768983907.1663668573.

[48] Borcsa M., Pomini V. (2017). Virtual Relationships and Systemic Practices in the Digital Era. Contemp. Fam. Ther..

[49] Hertlein K.M., Blumer M.L.C. (2013). The Couple and Family Technology Framework: Intimate Relationships in a Digital Age.

[50] Garb H.N. (2007). Computer-administered interviews and rating scales. Psychol. Assess..

[51] Hyler S.E., Gangure D.P., Batchelder S.T. (2005). Can telepsychiatry replace in-person psychiatric assessments? A review and meta-analysis of comparison studies. CNS Spectr..

[52] Schopp L., Johnstone B., Merrell D. (2000). Telehealth and neuropsychological assessment: New opportunities for psychologists. Prof. Psychol. Res. Pract..

[53] Singh S.P., Arya D., Peters T. (2007). Accuracy of telepsychiatric assessment of new routine outpatient referrals. BMC Psychiatry.

[54] Luxton D.D., Pruitt L.D., Osenbach J.E. (2014). Best practices for remote psychological assessment via telehealth technologies. Prof. Psychol. Res. Pract..

[55] Grady B., Myers K.M., Nelson E.L., Belz N., Bennett L., Carnahan L., Decker V.B., Holden D., Perry G., Rosenthal L. (2011). Evidence-based practice for telemental health. Telemed. e-Health.

[56] Ruskin P.E., Reed S., Kumar R., Kling M.A., Siegel E., Rosen M., Hauser P. (1998). Reliability and acceptability of psychiatric diagnosis via telecommunication and audiovisual technology. Psychiatr. Serv..

[57] Shore J.H., Savin D., Orton H., Beals J., Manson S.M. (2007). Diagnostic reliability of telepsychiatry in American Indian veterans. Am. J. Psychiatry.

[58] Lexcen F.J., Hawk G.L., Herrick S., Blank M.B. (2006). Use of Video Conferencing for Psychiatric and Forensic Evaluations. Psychiatric Serv..

[59] Manguno-Mire G.M., Thompson J.W., Shore J.H., Croy C.D., Artecona J.F., Pickering J.W. (2007). The use of telemedicine to evaluate competency to stand trial: A preliminary randomized controlled study. J. Am. Acad. Psychiatry Law..

[60] Cullum C.M., Weiner M.F., Gehrmann H.R., Hynan L.S. (2006). Feasibility of telecognitive assessment in dementia. Assessment.

[61] Galusha-Glasscock J.M., Horton D.K., Weiner M.F., Cullum C.M. (2016). Video Teleconference Administration of the Repeatable Battery for the Assessment of Neuropsychological Status. Arch. Clin. Neuropsychol..

[62] Grosch M.C., Weiner M.F., Hynan L.S., Shore J., Cullum C.M. (2015). Video teleconference-based neurocognitive screening in geropsychiatry. Psychiatry Res..

[63] Harrell K.M., Wilkins S.S., Connor M.K., Chodosh J. (2014). Telemedicine and the evaluation of cognitive impairment: The additive value of neuropsychological assessment. J. Am. Med. Dir. Assoc..

[64] Loh P.K., Donaldson M., Flicker L., Maher S., Goldswain P. (2007). Development of a telemedicine protocol for the diagnosis of Alzheimer's disease. J. Telemed. Telecare.

[65] Temple V., Drummond C., Valiquette S., Jozsvai E. (2010). A comparison of intellectual assessments over video conferencing and in-person for individuals with ID: Preliminary data. J. Intellect. Disabil. Res..

[66] Turkstra L.S., Quinn-Padron M., Johnson J.E., Workinger M.S., Antoniotti N. (2012). In-person versus telehealth assessment of discourse ability in adults with traumatic brain injury. J. Head Trauma Rehabil..

[67] Wadsworth H.E., Dhima K., Womack K.B., Hart J., Weiner M.F., Hynan L.S., Cullum C.M. (2018). Validity of Teleneuropsychological Assessment in Older Patients with Cognitive Disorders. Arch. Clin. Neuropsychol..

[68] Sammons M.T., VandenBos G.R., Martin J.N. (2020). Psychological Practice and the COVID-19 Crisis: A Rapid Response Survey. J. Health Serv. Psychol..

[69] Perle J.G., Langsam L.C., Randel A., Lutchman S., Levine A.B., Odland A.P., Nierenberg B., Marker C.D. (2013). Attitudes toward psychological telehealth: Current and future clinical psychologists' opinions of internet-based interventions. J. Clin. Psychol..

[70] Batterham P.J. (2014). Recruitment of mental health survey participants using Internet advertising: Content, characteristics and cost effectiveness. Int. J. Methods Psychiatr. Res..

[71] Humer E., Stippl P., Pieh C., Pryss R., Probst T. (2020). Experiences of Psychotherapists with Remote Psychotherapy During the COVID-19 Pandemic: Cross-sectional Web-Based Survey Study. J. Med. Internet Res..

[72] Bethlehem J. (2010). Selection Bias in Web Surveys. Int. Stat. Rev..

[73] WMA (2013). World Medical Association Declaration of Helsinki: Ethical principles for medical research involving human subjects. JAMA.

