# Supplementary material for: Attitudes of Mental Health Professionals towards Telepsychology during the Pandemic: A Pilot Study
Source: Healthcare (Basel). 2023 May 25;11(11):1542. doi: 10.3390/healthcare11111542 (PMC10253044; doi:10.3390/healthcare11111542)
Supplement: Supplementary file 1 [file healthcare-11-01542-s001.zip › healthcare-2338571-supplementary.pdf]

ONLINE PSYCHOLOGICAL ASSESSMENT AND TREATMENT

We are asking you to participate in a research study on online psychological assessment and treatment.
The aim of the study is to investigate critical issues and salient aspects of online psychological therapies aimed at mental well-being. Your contribution will be fundamental and valuable to achieve this aim.

This short questionnaire is intended exclusively for PSYCHOTHERAPISTS or PSYCHOLOGISTS who have had experience administering online psychological treatment.

The estimated time to complete the questionnaire is about 12 minutes.

This project has granted approval of the Ethics Commission of the University.
Data will be treated in an anonymous and aggregate form.
For further information, please contact us by email.

Thank you very much for your cooperation.

* Required

Informed consent	Research title: Online psychological assessment and treatment.<br><br>I declare that I am aware that:<br>- each participant is free to ask for clarification of the data collection procedure;<br>- each participant is free to abandon the research at any time;<br>- refusal to participate does not entail any negative consequences for the participant;<br>- the personal data collected will not be passed on to persons not directly involved in the research;<br>- the personal data collected will be processed anonymously;<br>- the results will be presented in an aggregate form and with every precaution necessary to avoid identifiability of the participants;<br>- the research is conducted in compliance with the Code of Ethics of the Italian Psychological Association (http://www.aipass.org/files/Codice_Etico_AIP.pdf).<br><br>Your data will be processed as provided for in the Privacy Regulations, in accordance with Legislative Decree 101/2018 adapting the Personal Data Protection Code (Legislative Decree 196/2003) to the provisions of EU Regulation 2016/679 (GDPR). The processing of data collected as part of the research, their communication to third parties, and/or their publication for scientific purposes are allowed, but can only take place after the data have been made anonymous.<br><br>I also declare<br>- I am of legal age;<br>- I have carefully read all the points of the declaration;<br>- I give my consent to participate in the research.

1. I declare that I have read the text of the informed consent and give/do not give my consent *

Mark only one oval.

- I consent
- I do not consent

Online experience

2. Do you have experience administering psychological treatments ONLINE? *

Mark only one oval.

- Yes
- No
  Skip to section 13 (Appreciation)

Professional profile

3. Please select your professional qualification: *

Mark only one oval.

- Psychologist
- Psychologist-psychotherapist
- Medic-psychotherapist

4. Select your orientation (therapeutic approach): *

Mark only one oval.

- ⬭ CBT
- ⬭ Psychodynamic
- ⬭ Psychoanalytic
- ⬭ Relational systemic
- ⬭ Integrated
- ⬭ Humanistic
- ⬭ Gestalt therapy
- ⬭ Other: ____________

5. Do you have specific training in neuropsychology? *

Check all that apply.

- ☐ No
- ☐ 1st or 2nd level master's degree
- ☐ School of specialisation
- ☐ PhD
- ☐ Other: ____________

6. Please select your prevailing work context: *

Mark only one oval.

- Private
- Public
- Affiliated
- Other: ____________

7. Select your main job position: *

Mark only one oval.

- Private professional
- Employee
- Other: ____________

Personal data

8. Age: *

Mark only one oval.

- <30
- 30-39
- 40-49
- 50-59
- 60-69
- >69

9. Gender: *

Mark only one oval.

- F
- M
- Non binary
- Other: ____________

10. Please select your country of residence: *

Mark only one oval.

- Australia
- Austria
- Belgium
- Brazil
- Canada
- Denmark
- Finland
- France
- Germany
- Greece
- Greenland
- Ireland
- Israel
- Italy
- Norway
- Portugal
- United Kingdom
- Spain
- United States of America
- Sweden
- Switzerland
- Other: ____________

11. Which category does the city you live in belong to (in relation to population density)? *

Mark only one oval.

- ⬭ Large cities and metropolises (high population density)
- ⬭ Medium/small cities and suburbs (medium population density)
- ⬭ Rural areas (low population density)
- ⬭ Other: ____________

Clinical experience

12. Please select how many years of clinical experience you have: *

Mark only one oval.

- ⬭ <6
- ⬭ 6-10
- ⬭ 11-20
- ⬭ 21-30
- ⬭ 31-40
- ⬭ >40

13. Select how many years of clinical experience you have ONLINE: *

Mark only one oval.

- <1 anno
- 1-3 anni
- 4-6 anni
- 7-9 anni
- >9 anni

14. How many clients/patients do you receive IN PERSON per week? *

Mark only one oval.

- None
- 1-10
- 11-20
- 21-30
- >30

15. How many customers/patients do you receive ONLINE per week? *

Mark only one oval.

- 1-5
- 6-10
- 11-20
- 21-30
- >30

16. What are your motivations for doing psychological treatment ONLINE? *

Check all that apply.

- [ ] Geographical distances
- [ ] Time saving
- [ ] Prevention of COVID-19 infection
- [ ] Saving of expenses related to running a private practice
- [ ] Other: ____________________

17. The majority of your customers/patients ONLINE are: *

Mark only one oval.

- ⬭ People you have seen from the first time only online
- ⬭ Previous clients/patients who switched from face-to-face to online mode
- ⬭ People who attend in mixed mode (in person alternating with online)
- ⬭ Other: ____________

18. Among the customers/patients you receive ONLINE are: *

Mark only one oval per row.

	Yes	No
**Children**	⬭	⬭
**Adolescents**	⬭	⬭
**Adults**	⬭	⬭
**Elderly**	⬭	⬭

19. What types of counselling/therapies do you carry out ONLINE? *

Mark only one oval per row.

	Yes	No
**Individuals**	◯	◯
**Couple**	◯	◯
**Family**	◯	◯
**Group**	◯	◯

20. Your ONLINE psychology treatments concern: *

Mark only one oval per row.

	Yes	No
**Anxiety disorders**	◯	◯
**Depressive disorders**	◯	◯
**PTSD**	◯	◯
**Cognitive disorders**	◯	◯
**Other**	◯	◯

21. How useful do you think having previous clinical experience in the field is for success in online practice? *

Mark only one oval.

	1	2	3	4	5	
Not at all	◯	◯	◯	◯	◯	Very much

Online setting

22. Have you ever been confronted with the following problems in the ONLINE setting? *

Mark only one oval per row.

	Yes	No
**Audio/video problems**	⬭	⬭
**Internet connection problems**	⬭	⬭
**Do not hear or see properly**	⬭	⬭
**Interruptions**	⬭	⬭
**Privacy issues**	⬭	⬭
**Failure to convey empathy**	⬭	⬭
**More distractions**	⬭	⬭
**Difficulties in finding a professional space**	⬭	⬭
**Communication difficulties**	⬭	⬭
**Other**	⬭	⬭

23. Did you find advantages in the ONLINE setting (compared to face-to-face therapy)? *

Mark only one oval.

- No
- Yes

24. If yes, what are the most significant advantages of the ONLINE setting for you?

25. Did you find any criticalities in the ONLINE setting (compared to face-to-face therapy)? *

Mark only one oval.

- No
- Yes

26. If yes, what are the main critical issues you faced in the ONLINE setting?

____
____
____
____
____

27. Generally, the mental and physical fatigue you feel following ONLINE interviews compared to face-to-face interviews is? *

Mark only one oval.

- Lower
- Equal
- Major

28. Do you fear that you are exposed to more risks in the ONLINE setting than in the face-to-face setting? *

Mark only one oval.

- Yes
- No

29. If yes, what risks do you think you are most exposed to in ONLINE practice?

____

____

____

____

____

Online assessment and testing

30. Which remote test administration methods did you use? *

Check all that apply.

- [ ] Administration by phone call (synchronous)
- [ ] Administration by video call (synchronous)
- [ ] Administration via screen sharing (synchronous)
- [ ] Self-administration via platform (asynchronous)
- [ ] Other: ____________________

31. Have you ever administered the following tests ONLINE? *

Mark only one oval per row.

	Yes	No
**BAIBECK Anxiety Inventory**	⬭	⬭
**BDHIIBeck Depression Inventory-II**	⬭	⬭
**SAFA-D/m-sScale**	⬭	⬭
**SAFA-A/sScale**	⬭	⬭
**STAIState-Trait Anxiety Inventory - Forma Y**	⬭	⬭
**RCMAS-2-Revised Children's Manifest Anxiety Scale – Second Edition**	⬭	⬭
**PDSSPostpartum Despression Screening Scale**	⬭	⬭
**CDIChildren's Depression Inventory**	⬭	⬭
**SCL-90-RSymptom Checklist-90-R**	⬭	⬭
**MMPI-2 ONLINE Minnesota Multiphasic Personality Inventory -2**	⬭	⬭

32. Have you ever carried out an ONLINE neuropsychological assessment? *

Mark only one oval.

- Yes
- No

33. What advantages have you found in the ONLINE neuropsychological assessment?

34. What critical issues have you found in the ONLINE neuropsychological assessment?

New technologies

35. When you decided to start offering psychological treatment ONLINE, how did you train? *

Check all that apply.

- [ ] Requested advice from colleagues
- [ ] Requested professional advice
- [ ] Read guidelines
- [ ] Read scientific articles
- [ ] Read manuals
- [ ] Attended webinars
- [ ] Tutorials
- [ ] Attended an in-person training course
- [ ] Other: ____________

36. Based on your experience, do you think that academic and post-graduate training is adequate for the needs arising from ONLINE counselling, therapy, and assessment? *

Mark only one oval.

- ( ) Yes
- ( ) No

37. Based on your experience, do you think that the currently available further education courses are adequate for the needs arising from ONLINE counselling, therapy, and assessment? *

Mark only one oval.

- Yes
- No

38. When you decided to start your ONLINE activity, did you receive support and guidance from your professional community (order/association)? *

Mark only one oval.

- Yes
- No

39. Which devices do you prefer to use for ONLINE therapy? *

Check all that apply.

- [ ] Smartphone
- [ ] Tablet
- [ ] PC
- [ ] Smartwatch
- [ ] Other: ______________

40. Which modality do you mainly use with your clients/patients in your ONLINE psychological treatments? *

Check all that apply.

- [ ] Videoconference
- [ ] Mail
- [ ] SMS
- [ ] Chat
- [ ] Telephone calls
- [ ] Mixed mode
- [ ] Other: ____________

41. Which of these software and videoconferencing services do you prefer to use for your ONLINE clinical practice? *

Check all that apply.

- [ ] Skype
- [ ] GoToMeeting
- [ ] Teams
- [ ] Zoom
- [ ] Webex
- [ ] WhatsApp
- [ ] FaceTime
- [ ] Google Meet
- [ ] Other: ____________

42. What are the features that made you prefer one videoconferencing software over another? *

Check all that apply.

- [ ] Ease of use
- [ ] Possibility of screen sharing
- [ ] Adequate cost
- [ ] Ease of formulating access links
- [ ] Most suitable for group videoconferencing
- [ ] I know it better than others
- [ ] The choice was made by my organisation
- [ ] Other: ____________

43. What kind of connection do you use to carry out psychological assessments and treatments ONLINE? *

Mark only one oval.

- ( ) Ultra broadband
- ( ) Broadband
- ( ) ADSL
- ( ) Internet via satellite
- ( ) 5G cellular data network
- ( ) 4G cellular data network
- ( ) 3G cellular data network
- ( ) Other: ____________

44. Do you have a website for your professional activity? *

Mark only one oval per row.

	Yes	No
**Personal website**	◯	◯
**Company website**	◯	◯

45. On which digital platforms for psychologists can your potential clients/patients contact you? *

Check all that apply.

- [ ] None
- [ ] ProntoPro
- [ ] Unobravo
- [ ] Liviconnect
- [ ] Psicologo4you
- [ ] PsicologiOnline
- [ ] Psiqo
- [ ] GuidaPsicologi
- [ ] Tokitus Online Therapy
- [ ] BetterHelp
- [ ] Talkspace
- [ ] Other: ____________

46. On which social networks can your clients/patients find you with a professional profile? *

Check all that apply.

- [ ] Facebook
- [ ] Instagram
- [ ] LinkedIn
- [ ] Twitter
- [ ] YouTube
- [ ] Other: ____________________

47. How can clients/patients make an ONLINE appointment with you? *

Check all that apply.

- [ ] Telephone call
- [ ] email
- [ ] SMS
- [ ] Chat
- [ ] Online agenda
- [ ] Other: ____________________

Fees for services

48. Compared to that applied to in-person sessions, your fee for ONLINE sessions is: *

Mark only one oval.

- Higher
- Equal
- Lower

49. Do you think there should be a minimum fee for ONLINE sessions? *

Mark only one oval.

- Yes
- No

50. Receives compensation for services ONLINE: *

Mark only one oval.

- In advance
- At the end of the session
- After the session
- Through the organisation I work for
- Other: ____________

51. Considers that the risk of non-compliance in ONLINE therapies, compared to face-to-face therapies, is: *

Mark only one oval.

- Major
- Equal
- Lower

Prospects

52. How satisfied are you with the possibility of doing clinical work ONLINE? *

Mark only one oval.

	1	2	3	4	5	
Not at all						Very much

53. Do you have doubts about the effectiveness of ONLINE therapies compared to face-to-face therapies? *

Mark only one oval.

- Yes
- No

54. Considers that therapeutic adherence in ONLINE practices compared to face-to-face therapy is: *

Mark only one oval.

- Lower
- Equal
- Greater

55. Do you think that the drop-out rate of a client/patient from ONLINE therapy can be significantly higher than in-patient therapy? *

Mark only one oval.

- Yes
- Don't know
- No

56. Do you plan to continue with ONLINE psychological therapies in the future? *

Mark only one oval.

- Yes
- Don't know
- No

57. What investments do you think your scientific community should make to foster the development of ONLINE clinical practices?

____
____
____
____
____

58. In the future, what do you think you will personally invest in to improve your ONLINE clinical practice?

____
____
____
____
____

59. Would you be willing to be contacted for a possible short in-depth interview? *

Mark only one oval.

- ( ) Yes
- ( ) No
  Skip to section 13 (Appreciation)

Contact details

60. FIRST AND LAST NAMES *

61. EMAIL *

62. TELEPHONE NUMBER

Appreciation

Thank you very much for your participation.

We would be grateful if you would share this questionnaire with your colleagues.

YOU MAY NOW PRESS SUBMIT TO FINISH.

This content is neither created nor endorsed by Google.

Google Forms

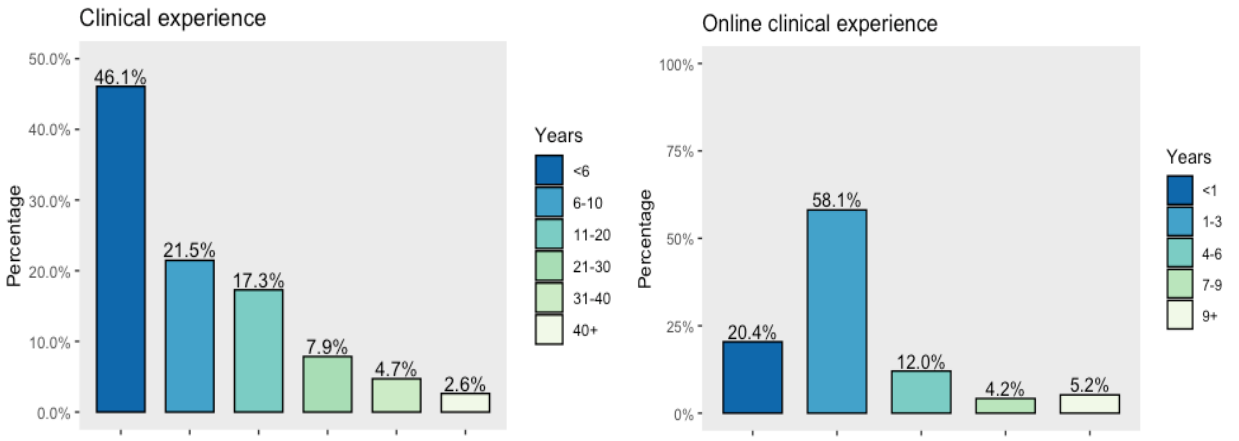

(**a**) (**b**)

Figure S1. Years of clinical experience (**a**) and years of online clinical experience (**b**) accrued by the sample of mental health professionals.

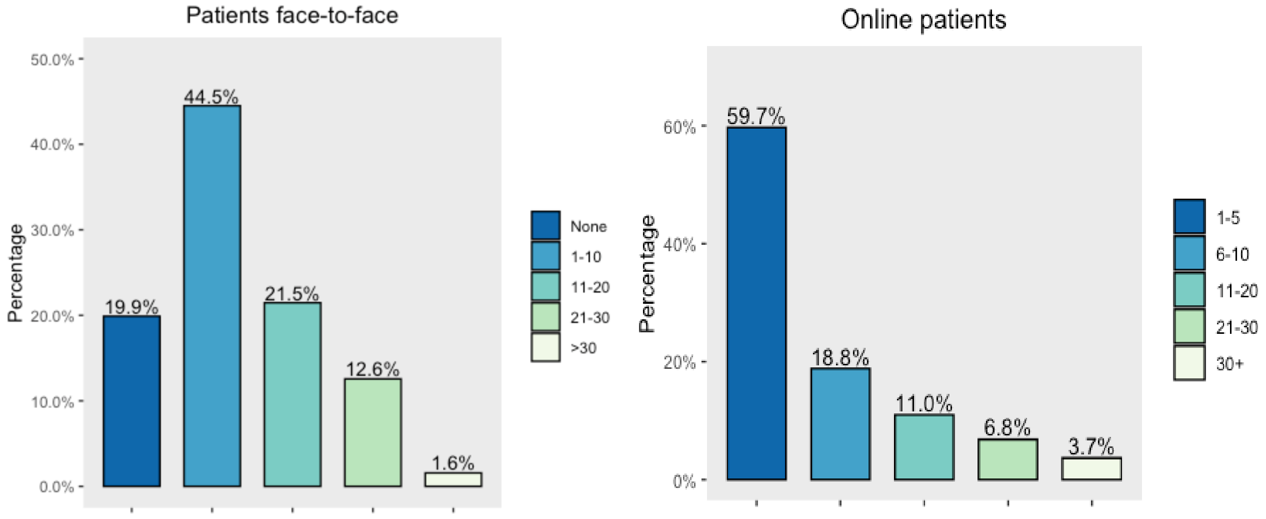

(**a**) (**b**)

Figure S2. Distributions of patients received face-to-face (**a**) per week and patients received online (**b**) per week by the sample of mental health professionals.

Table S1. Frequency distribution – health professionals' country of residence.

	N	%
Australia	2	~1
Colombia	1	~0.5
France	1	~0.5
Greece	2	~1
Guyana	1	~0.5
India	2	~1
Iran	1	~0.5
Israel	1	~0.5
Italy	139	~73
Lebanon	2	~1
Malaysia	1	~0.5
Portugal	1	~0.5
Spain	1	~0.5
UK	11	~5.7
USA	25	~13

Table S2. Descriptive statistics about the new technologies

**Variables**	***N***	**%**
Which modality do you mainly use with your clients/patients in your ONLINE psychological treatments?*		
Videoconference	178	93
E-mail	23	12
Chat	29	15
Telephone calls	53	28
Mixed mode	23	12
Which software and videoconferencing services do you prefer to use for your ONLINE clinical practice:*		
Doxy	6	3
Teams	17	9
Zoom	78	41
Facetime	29	15
Skype	124	65
WhatsApp	80	42
Google Meet	61	32
What are the features that made you prefer one videoconferencing software over another?*		
Ease of use	166	87
Possibility of screen sharing	48	25
Adequate cost	50	26
Ease of formulating access links	67	35
Most suitable for group videoconferencing	19	10
I know it better than others	59	31
The choice was made by my organisation	23	12
On which social networks can your clients/patients find you with a professional profile?*		
Facebook	138	72
Instagram	139	73
Twitter	17	9
LinkedIn	134	70
YouTube	31	16
How can clients/patients make an ONLINE appointment with you?*		
Telephone call	166	87
E-mail	157	82
SMS	101	53
Chat	99	52
Online agenda	38	20

Note. *The asterisk indicates that multiple answers were possible per respondent.

Table S3.

Pearson's Chi-squared test	
X-squared	16.844
df	18
p-value	0.5339

We were also interested in investigating the presence of a possible association between the degree of satisfaction in carrying out online therapy (item 52) and therapeutic approach. The Chi-squared test showed that there was no association between the two variables. This indicates that there was no discrimination between therapeutic approach and perceived satisfaction among the sample of mental health professionals.
